# Insights into the role of non-coding RNAs in the development of insecticide resistance in insects

**DOI:** 10.3389/fgene.2024.1429411

**Published:** 2024-07-05

**Authors:** Huamei Xiao, Chunhui Ma, Ruizhi Peng, Meiqiong Xie

**Affiliations:** Key Laboratory of Crop Growth and Development Regulation of Jiangxi Province, College of Life Sciences and Resource Environment, Yichun University, Yichun, China

**Keywords:** insects, insecticide resistance, ncRNAs, ceRNA network, ncRNA regulation

## Abstract

Pest control heavily relies on chemical pesticides has been going on for decades. However, the indiscriminate use of chemical pesticides often results in the development of resistance in pests. Almost all pests have developed some degree of resistance to pesticides. Research showed that the mechanisms of insecticide resistance in insects encompass metabolic resistance, behavioral resistance, penetration resistance and target-site resistance. Research on the these mechanisms has been mainly focused on the *cis*-regulatory or *trans*-regulatory for the insecticide resistance-related genes, with less attention paid to non-coding RNAs (ncRNAs), such as microRNA (miRNA), long non-coding RNA (lncRNA), and circular RNA (circRNA). There has been increased studies focus on understanding how these ncRNAs are involved in post-transcriptional regulation of insecticide resistance-related genes. Besides, the formatted endogenous RNA (ceRNA) regulatory networks (lncRNA/circRNA-miRNA-mRNA) has been identified as a key player in governing insect resistance formation. This review delves into the functions and underlying mechanisms of miRNA, lncRNA, and circRNA in regulating insect resistance. ncRNAs orchestrate insect resistance by modulating the expression of detoxification enzyme genes, insecticide target genes, as well as receptor genes, effectively regulating both target-site, metabolic and penetration resistance in insects. It also explores the regulatory mechanisms of ceRNA networks in the development of resistance. By enhancing our understanding of the mechanisms of ncRNAs in insecticide resistance, it will not only provide valuable insights into the new mechanisms of insecticide resistance but also help to enrich new directions in ncRNAs gene regulation research.

## 1 Introduction

Pests globally cause a significant reduction, estimated to be between 10% and 30% in annual food production, resulting in an economic loss of approximately $20 billion (Pimentel et al., 2000; Oerke, 2006). Pesticides are considered the primary and efficient method of directly controlling pests, with an annual usage surpassing one billion kilograms and an upward trend in specific countries (Alavanja, 2009). Nevertheless, the extensive use of insecticides has resulted in varying levels of resistance in over 600 insect species (Connor et al., 2011). Particularly, the diamondback moth (*Plutella xylostella*, DBM) exhibited the highest level of resistance, being resistant to 101 insecticides with different active ingredients. Additionally, other common pests in vegetables and rice crops, including *Bemisia tabaci*, *Aphis gossypii* Glover, and the rice stem bore *Chilo suppressalis*, demonstrated resistance to 69, 52, and 39 insecticides, respectively (APRD, 2024). The simultaneous application of new and traditional pesticides in agriculture frequently hastens the development of resistance to newer pesticides (Sayyed and Wright, 2006). Insecticide resistance has been a common and intractable problem in the world’s agricultural production. Furthermore, the use of new crop protection chemistries faces challenges due to concerns regarding environmental safety, human health, and the escalating costs associated with their discovery, development, and registration. Therefore, there is an urgent requirement for prudent pesticide utilization and a thorough comprehension of the mechanisms that drive insect resistance to pesticides (R4P Network, 2016).

The research conducted in the last few decades has revealed that insecticide resistance in insects involves various mechanisms, including metabolic resistance, behavioral resistance, penetration resistance, target-site resistance and sequestration resistance (Siddiqui et al., 2022; Pu and Chung, 2024). Indeed, research on insecticide resistance has traditionally focused on protein-coding genes, particularly mutations or changes in the insecticide target genes and increased expression of detoxification enzyme genes (Siddiqui et al., 2022). In other words, research has focused on the *cis*-regulatory or *trans*-regulatory for these genes. However, biological processes are regulated by not only protein-coding genes but also non-coding RNAs (ncRNAs). In recent years, there has been an increased focus on studying how ncRNAs involved in posttranscriptional regulation of insecticide resistance-related genes can impact resistance. These studies aim to unravel the mechanisms by which ncRNAs influence the expression and function of genes involved in insecticide resistance and explore their potential contributions to resistance mechanisms. By considering the regulatory role of ncRNAs, researchers hope to gain a more comprehensive understanding of the molecular processes underlying insecticide resistance (Pu and Chung, 2024).

ncRNAs have been shown to target both DNA and RNA substrates, modulating signaling pathways involved in critical biological processes such as development and differentiation (Muthu Lakshmi Bavithra et al., 2023). Insects harbor a large number of ncRNA genes, and among them, microRNA (miRNA) has emerged as a crucial regulator in their life processes, impacting key aspects including metamorphosis, reproduction, immune responses, and the detoxification of plant toxic substances (Zhang et al., 2021b). Moreover, long non-coding RNA (lncRNA) actively participates in insect development, innate immunity, and chromosome dosage compensation processes by coordinating gene expression at epigenetic, transcriptional, and post-transcriptional levels (Li et al., 2019). Circular RNA (circRNA), a relatively novel molecule, also contributes to the regulatory landscape, influencing the development, reproduction, and innate immune responses of insects (Muthu Lakshmi Bavithra et al., 2023). Furthermore, ncRNAs, particularly the lncRNA/circRNA-miRNA-mRNA (competing endogenous RNAs, ceRNAs) regulatory network, has been identified as a key player in governing the formation of insect resistance, offering a potential strategy for pest control (Muthu Lakshmi Bavithra et al., 2023). LncRNAs and circRNAs can indeed function as ceRNA by sequestering miRNAs, thereby relieving the inhibition of detoxification enzyme genes and target genes. This regulatory mechanism plays a crucial role in shaping the metabolic and target-site resistance observed in insects. Additionally, lncRNAs have been discovered to serve as miRNA sponges, regulating the expression of cuticle proteins. This modulation, in turn, modulating insect penetration resistance to insecticides by regulating epidermal thickness (Pu and Chung, 2024).

With the progress of next-generation sequencing technologies and bioinformatics tools, researchers now have the capability to investigate the molecular basis of insecticide resistance. This includes the analysis of diverse regulators and modifiers of gene expression levels, such as miRNA, lncRNA, and circRNA, as well as the networks that form among these regulators and protein-coding genes (Pu and Chung, 2024). The primary focus of this review is to investigate the intricate mechanisms by which miRNA, lncRNA, and circRNA influence the development of insect resistance against various insecticides. Based on the findings, it will highlight the multifaceted roles of lncRNAs and circRNAs serving as ceRNA in orchestrating insect resistance mechanisms and provide insights into potential strategies for managing and combating insecticide resistance.

## 2 Types of non-coding RNAs

The ncRNAs can be classified into different categories according to their length and intended function. These categories include ribosomal RNA (rRNA), transfer RNA (tRNA), lncRNAs, small ncRNAs (sncRNAs) such as miRNA, piwi-interacting RNA (piRNA) and small nuclear RNA (snRNA), as well as a newly class of ncRNA known as circRNA (Zhang et al., 2019).

MiRNA, a class of endogenous small RNA molecules ranging from 22 to 25 nucleotides in length, plays a crucial role in the post-transcriptional regulation of genes. It exerting its influence by negatively regulating target genes through mRNA cleavage or inhibiting the translation of target proteins, ultimately leading to gene silencing (Bartel, 2004; Bartel, 2005). The advancement of miRNA sequencing technologies has facilitated the discovery of a multitude of miRNAs in insects. The InsectBase 2.0 database contains an impressive 112,162 mature miRNA sequences from 807 species, highlighting the widespread prevalence and functional diversity of miRNAs in the insect kingdom (Mei et al., 2022).

LncRNAs are a class of non-protein-coding RNAs that are typically longer than 200 nucleotides in length. Unlike protein-coding genes, lncRNA genes lack long open reading frames. However, they undergo a similar processing mechanism as protein-coding genes. Transcribed by RNA polymerase II and subjected to splicing, they acquire a 5′ cap structure and a 3′ polyA tail, culminating in a gene structure resembling mRNA (Guttman et al., 2009). It is now recognized as a widely distributed element in eukaryotes, playing a pivotal role in regulating gene expression (Hung and Chang, 2010; Rinn and Chang, 2012; Batista and Chang, 2013). The InsectBase 2.0 database contains a vast array of 1,293,430 lncRNA sequences from 376 species, attesting to the widespread prevalence of lncRNA in the insect kingdom (Mei et al., 2022).

CircRNAs, a recently discovered class of non-coding RNAs, distinguish themselves from linear counterparts like miRNAs and lncRNAs due to their unique covalently closed-loop structure. This distinctive structure, formed through circularization, splicing, and shearing of parental mRNA precursor (pre-mRNA), results in a closed loop where the 5′ and 3′ ends of the pre-mRNA target sequences are connected. Unlike linear RNA molecules, circRNAs lack the 5′ cap and 3′ poly(A) tail, making them resistant to degradation by RNA exonucleases. As a result, circRNAs exhibit a longer half-life and greater stability (Cocquerelle et al., 1992; Capel et al., 1993; Chen, 2020). CircRNAs can originate from the exons, introns and intergenic sequences of coding genes (Salzman, 2016; Kristensen et al., 2019). Their involvement in various biological processes and signaling pathways is primarily achieved through the regulation of gene transcription (Li et al., 2021e), miRNA adsorption (Xu et al., 2018), binding to RNA binding proteins (RBPs) (Xia et al., 2018) and encoding polypeptides (Chen and Sarnow, 1995).

Non-coding RNAs play a crucial roles in the development of insecticide resistance (Pu and Chung, 2024). In this review, the focus is on three types of ncRNAs: miRNAs, lncRNAs, circRNAs. Each of these ncRNAs has been found to regulate the development of insecticide resistance in insects. Moreover, there is evidence of a regulatory network involving lncRNA/circRNA-miRNA-mRNA that is involved in regulating insect resistance to pesticides.

## 3 The regulatory role of miRNAs in insecticide resistance development

### 3.1 The mechanisms of insecticide resistance in insects

The mechanisms of insecticide resistance in insects encompass metabolic resistance, behavioral resistance, penetration resistance, target-site resistance (Siddiqui et al., 2022), sequestration resistance (Pu and Chung, 2024), and other types. However, the majority of studies in this field have predominantly focused on the target-site resistance and metabolic resistance.

Mutations and duplications in the acetylcholinesterase gene and mutations in the genes encoding insecticide target receptors, such as voltage-gated sodium channels, chloride channels, nicotinic acetylcholine receptors, ryanodine receptor, and octopamine receptors, can lead to target-site resistance in insects. Mutations in chitin syntheses. Furthermore, mutations in chitin synthesis genes and lipid biosynthesis enzymes are also implicated in target-site resistance. (Siddiqui et al., 2022). Regarding resistance to *Bacillus thuringiensis* (Bt) Cry proteins, the main mechanism observed is the mutation or down-regulation of the expression of midgut receptor-binding protein genes such as cad, ALP, APN, and ABC transporter proteins (Jurat-Fuentes et al., 2021). This mechanism allows insects to resist the toxic effects of Bt Cry proteins.

Detoxification enzyme systems play a crucial role in insecticide resistance to metabolizinge pesticides into compounds of less toxic or non-toxic compounds (Nauen et al., 2022). In resistant populations, these systems can be enhanced through various mechanisms. Mutations and amplifications of detoxification enzymes, activation of related regulatory factors, and over-expression of detoxification enzymes can all contribute to increased detoxification capabilities. Several enzyme systems have been implicated in insecticide detoxification, including carboxylesterases (CarE), glutathione S-transferases (GSTs), Cytochrome P450 monooxygenases (P450s), ABC and UDP glucosyl transferase (UGT) (Karunaratne and Surendran, 2022). Resistant populations may possess an abundance of these enzymes or enzyme systems with heightened activity, allowing them to efficiently metabolize a broader spectrum of pesticides (Vyas et al., 2023). Specific mutations in detoxification enzymes can also lead to resistance. For example, the F116V mutation in the substrate recognition region SRS1 of the CYP9A186 enzyme in the avermectin-resistant strain of the beet armyworm enables the insect to detoxify and metabolize avermectin, contributing to pest resistance (Zuo et al., 2021). In some cases, resistance can be linked to signaling pathways and gene expression. Activation of the *ERK* and *p38* signals of the serine kinase signaling pathway MAPK in tobacco whitefly phosphorylates the serine 111 of the CREB protein, leading to the activation of the downstream target gene *CYP6CM1*. This activation results in the over-expression of *CYP6CM1*, which enhances the metabolism of insecticides and ultimately leads to neonicotinoid resistance in tobacco whitefly (Yang et al., 2020). Furthermore, mutations in the 5′-UTR region of the resistance gene *CYP4C64* have been associated with thiamethoxam resistance in insects. These mutations cause the gene to be over-expressed, leading to resistance to the neonicotinoid insecticide thiamethoxam (Yang et al., 2021b).

Absolutely, insects can also develop penetration resistance as a defense mechanism to reduce the entry of insecticides into their bodies. By creating a barrier using their outer epidermis, insects can protect themselves from a wide range of chemical pesticides. This mechanism is associated with the up-regulation of the expression of CYP genes, which in turn alter the composition of cuticular hydrocarbons (CHCs) in the insect epidermis (Pu and Chung, 2024). For instance, in the mosquito species *Anopheles gambiae*, changes in the CYP4G enzyme can alter the composition of CHCs in the insects’ epidermis. This alteration accelerates the synthesis of CHCs, leading to a thickening of the epidermis. As a result, the amount of insecticides penetrating into the body is reduced, thereby conferring resistance to deltamethrin. This penetration resistance mechanism has also been observed in other insect species such as field whitefly and *Blattella germanica* (Chen et al., 2020; Liang et al., 2022).

In addition to those well-known modes of resistance, a newly emerged mode called sequestration resistance has been identified. Sequestration resistance occurs when olfactory proteins such as chemosensory proteins (CSPs) and odorant-binding proteins (OBPs), are over-expressed in certain tissues, such as the legs of insects. This over-expression allows the insect to develop resistance by binding the insecticide to these proteins and sequestering it before the insecticide reaches its target protein (Pu and Chung, 2024).

### 3.2 Involvement of miRNAs in the regulation of metabolic resistance

Metabolic resistance involves the enzymatic degradation or sequestration of insecticides by insects, constituting a critical detoxification mechanism that diminishes their toxic effects. miRNAs have been demonstrated to precisely regulate the expression of enzymes implicated in detoxification pathways, thereby affecting the metabolic fate of insecticides (Muthu Lakshmi Bavithra et al., 2023). Given that miRNA can exert negative regulation on target genes, miRNA-mediated regulation of a detoxification metabolic enzyme can lead to reduced expression or inhibited translation. Consequently, the enzyme’s capacity to metabolize pesticides decreases, resulting in heightened sensitivity to these substances. Consequently, the expression levels of miRNAs targeting detoxification metabolic enzymes can be precisely regulated, exhibiting a negative correlation with resistance levels (Zhang et al., 2021b). Our findings emphasize that miRNAs target diverse insect detoxification enzymes, modulate the expression of target genes, and accordingly regulate insect metabolic resistance to different insecticides (Table 1).

**TABLE 1:** List of insect microRNAs involved in metabolic resistance to different insecticides.

Species	MicroRNA	Target gene	miRNA regulation	Exposure	Year	References
*Culex pipienspallens*	miR-71	*CYP325BG3*	Down	Deltamethrin	2014	Hong et al. (2014)
	miR-278-3p	*CYP6AG11*			2015	Lei et al. (2015)
	miR-2	*CYP9J35*			2019	Guo et al. (2017)
		*CYP325BG3*				
	miR-4448	*CYP4H31*			2021	Li et al. (2021c)
	miR-13664	*CYP314A1*			2019	Sun et al. (2019)
	miR-932	*CPR5*	Up		2016	Liu et al. (2016)
	miR-92a	*CPR4*			2017	Ma et al. (2017)
	miR-285	*CYP6N23*			2016	Tian et al. (2016)
	miR-13	*CYP9J35*			2019	Fahmy et al. (2020)
	miR-279-3p	*CYP325BB1*			2021	Li et al. (2021d)
*Drosophila melanogaster*	miR-310-3p	*CYP6G1*	Down	DDT	2019	Seong et al., 2019; Seong et al., 2020
		*CYP6G2*				
	miR-311-3p	*CYP6G1*				
		*CYP6G2*				
	miR-312-3p	*CYP6G1*				
		*CYP6A8*				
	miR-313-3	*CYP6G1*				
		*CYP6G2*				
		*CYP4G1*				
		*CYP6A8*				
	miR-92a-3p	*CYP6G2*				
		*CYP4G1*				
*Plutella xylostella*	miR-8533-3p	*LCP-30*	Down	Chlorantraniliprole	2017	Zhu et al. (2017a)
	miR-8534-5p	*CYP6B6*				
	miR-375-5p	*CYP4G15*				
	miR-2b-3p	*CYP9F2*		Chlorantraniliprole Deltamethrin	2018	Etebari et al. (2018)
	miR-108	*JHE*	Up	Cry1Ac	2023	Yang et al. (2023)
	miR-234					
	miR-8528a	*GSTS1*		Chlorantraniliprole	2024	Liu et al. (2024)
*Spodoptera frugiperda*	miR-190-5p	*CYP6K2*	Down	Chlorantraniliprole	2022	Zhang et al. (2022a)
	miR-317-3p	*GST*		Indoxacarb	2024	Wu et al. (2024)
	miR-283-5p					
*Lymantria dispar*	novel-miR-4	*CYP4C1*		Cyantraniliprole	2023	Zhang et al. (2023)
*Sitobion miscanthi*	miR-316	*CYP4CJ6*	Up	Imidacloprid	2021	Zhang et al. (2021c)
*Nilaparvata lugens*	Novel_85	*CYP6ER1*	Down	Nitenpyram	2022	Mao et al. (2022)
	Novel_191	*CarE1*				
*Laodelphax striatellus*	PC-5p-30_205949	*CYP419A1*	Down	Triflumezopyrim	2023	Wang et al. (2023a)
	PC-3p-2522_840	*CYP6FL1*			2022	Yang et al. (2022b)
	PC-3p-446_6601	*GSTD2*				
		*UGT386F1*				
*Bemisia tabaci*	novel_miR-1517	*CYP6CM1*	Down	Imidacloprid	2023	Gong et al. (2023)
*Tetranychus cinnabarinus*	miR-1-3p	*TCGSTM4*	Down	Cyflumetofen	2018	Zhang et al. (2018)

P450s constitute a family of enzymes ubiquitously found across all life forms, catalyzing diverse oxidative transformations of both endogenous and exogenous substrates, and playing a pivotal role in detoxification processes (Nelson, 2018). Metabolic resistance development in insects is tightly associated with upregualted expression of P450 genes. However, recent studies have revealed that metabolic resistance in insects can also be linked to decreased expression of P450 genes, exemplified by *Varroa destructor* coumaphos-resistant populations. These populations evade organophosphorus toxicity by down-regulating the expression of the *CYP4EP4* gene, one of the mere 26 p450s in their genome. Consequently, this downregulation diminishes proinsecticide activation, ultimately leading to resistance against coumaphos (Vlogiannitis et al., 2021). *Culex pipiens pallens* emerges as a prominent pathogenic parasite accountable for malaria transmission. In the prevention and control of this parasite, pyrethroid agents such as deltamethrin play a pivotal role. The regulation of insect resistance to chemical pesticides by miRNA has been extensively studied. In the case of *C. pipiens*, miRNAs play a crucial role in governing the development of deltamethrin resistance by regulating P450 genes. In the deltamethrin-resistant strain of *C. pipiens*, there are five low-expressed miRNAs (miR-13664, miR-2, miR-71, miR-4448, and miR-278-3p) that individually target five P450 genes (*CYP314A1*, *CYP9J35*, *CYP325BG3*, *CYP4H31*, and *CYP6AG11*) (Hong et al., 2014; Lei et al., 2015; Guo et al., 2017; Sun et al., 2019; Li et al., 2021c). On the other hand, there are five highly expressed miRNAs (miR-92a, miR-285, miR-932, miR-13, and miR-279-3p) that respectively target *cytochrome P450 reductase (CPR) 4*, *CYP6N23*, *CPR5*, *CYP9J35*, and *CYP325BB1*, orchestrating the development of resistance to deltamethrin in *C. pipiens* (Liu et al., 2016; Tian et al., 2016; Ma et al., 2017; Fahmy et al., 2020; Li et al., 2021d). These studies highlight the intricate regulatory role of miRNAs in modulating the expression of specific P450 genes, ultimately influencing the development of resistance to deltamethrin in *C. pipiens.* In *D. melanogaster*, the metabolism of dichlorodiphenyltrichloroethane (DDT) is primarily associated with P450 gene. Low resistance to DDT is linked to the *CYP6G1* gene (Le Goff and Hilliou, 2017), while medium and high levels of DDT resistance involve multiple genes (Kim et al., 2018). *D. melanogaster* employs post-transcriptional regulation of target gene expression to detoxify DDT. This regulation includes the downregulation of miR-310-3p, miR-311-3p, miR-312-3p, miR-313-3p, and miR-92a-3p, which orchestrates the expression of *CYP6AG1*, *CYP6AG2*, *CYP6A8*, and *CYP4G1* (Seong et al., 2019; Seong et al., 2020). These findings demonstrate the intricate miRNA regulatory mechanisms involved in DDT metabolism and resistance in *D*. *melanogaster.*

Three low-expressed miRNAs (miR-8533-3p, miR-8534-5p, and miR-375-5p) in chlorantraniliprole-resistant strains of DBM target *LCP-30*, *CYP6B6*, and *CYP4G15* respectively, thereby regulating the high expression levels of these genes (Zhu et al., 2017b). Moreover, the downregulation of miR-2b-3p in DBM treated with chlorantraniliprole lead to the inhibition of the detoxification enzyme gene *CYP9F2*. Subsequent analysis involving both deltamethrin-resistant and -sensitive strains of DBM revealed that miR-2b-3p regulate the sensitivity to both chlorantraniliprole and deltamethrin through the modulation of *CYP9F2* gene expression (Etebari et al., 2018). The resistance of fall armyworm (*Spodoptera frugiperda*, FAW) to chlorantraniliprole is strongly linked to the regulation of *CYP6K2* gene expression by miR-190-5p, which binds to the 3′UTR of the *CYP6K2* gene. The injection of a miR-190-5p agomir results in a significantly reduction in *CYP6K2* gene expression and the sensitivity of FAW to chlorantraniliprole. Conversely, inhibiting miR-190-5p expression significantly increases the expression of *CYP6K2*, thereby enhancing the tolerance of FAW larvae to chlorantraniliprole (Zhang et al., 2022a). Additionally, miR-278-5p may be involved in resistance to the novel insecticide tetraniliprole (Zhang et al., 2022b). Moreover, miR-278-5p, miR-13b-3p, miR-10485-5p, and miR-10483-5p are potential contributors to resistance against cyantraniliprole and emamectin benzoate in FAW (Yang et al., 2021c). Downregulated novel-miR-4 targets *CYP4C1*, potentially influencing the sensitivity of *Lymantria dispar* to cyantraniliprole in cyantraniliprole-treated insects, based on the miRNA regulation patterns observed in DBM and FAW (Zhang et al., 2023). miRNA plays a critical role in regulating resistance, primarily through post-translational modification of CYP genes. For example, miR-316 exhibited differential downregulation in spirotetramat-resistant and -sensitive strains of *Sitobion miscanthi*. It binds to the 3′ UTR of *CYP4CJ6*, and inhibition of miR-316 expression leads to an increase in *CYP4CJ6* expression. This regulatory mechanism affects the sensitivity of *S. miscanthi* to spirotetramat (Zhang et al., 2021a). Additionally, a novel miRNA, novel_miR-1517, regulates the expression of *CYP6CM1* and influences the resistance of *B. tabaci* to imidacloprid (Gong et al., 2023). Planthoppers, such as *Nilaparvata lugens* and *Laodelphax striatellus*, are significant migratory pests that affect rice crops (Kil and Kim, 2023). They have developed resistance to imidacloprid, chlorpyrifos, deltamethrin, and other agents (Tang et al., 2022; Wen et al., 2022). In *N.lugens*, the low-expressed miRNA novel_85 can reverse-regulate the expression of *CYP6ER1* (Mao et al., 2022). Furthermore, the resistance of *L. striatellus* to triflumezopyrim involves miRNA PC-5p-30_205949 up-regulating the expression of *CYP419A1,* and PC-3p-2522_840 up-regulating *CYP6FL1* (Yang et al., 2022; Wang et al., 2023a).

CarE-mediated metabolic resistance in insects primarily results from the over-expression of the CarE gene and mutations in the encoded amino acid sequence (Cui et al., 2011). Juvenile hormone esterase (*JHE*) plays a crucial role in the metamorphosis of insects (Li et al., 2021b) and belongs to the CarE subfamily, one of eight subfamilies that regulate insect metabolic resistance (Ranson et al., 2002). Recent research reveals that miRNAs regulate the formation of resistance to Cry1Ac in DBM by influencing the expression of *JHE*. *PxJHE* is differentially expressed in Cry1Ac-resistant and -sensitive strains of DBM. Interfering with *PxJHE* expression increases the sensitivity of DBM to Cry1Ac. In the resistant strains, the highly expressed miRNAs miR-108 and miR-234 bind to the CDS region of *PxJHE*. Injection of miR-108 and miR-234 agomir significantly reduces the expression of *PxJHE*, thereby enhancing sensitivity to Cry1Ac. Conversely, inhibiting the expression of miR-108 or miR-234 increases *PxJHE* expression, reducing tolerance to Cry1Ac. Additionally, injection of miR-108 and miR-234 agomir results in reduced body length and weight, as well as a significant decrease in pupation rate of DBM (Yang et al., 2023). Furthermore, miRNA novel_191 was downregulated in mediating the resistance to nitenpyram in *N. lugens* by reverse regulating *CarE1* (Mao et al., 2022).

The detoxification enzyme GST utilizes multiple mechanisms to regulate the formation and development of insecticide resistance (Pavlidi et al., 2018). Pyrethroids, abamectin, and DDT can induce oxidative stress, leading to the formation of numerous peroxides upon entering the insect body. GST effectively eliminates these peroxides, thereby reducing their harmful effects on insects (Lumjuan et al., 2005; Liao et al., 2016; Li et al., 2021a). A highly expressed GST gene, *PxGSTS1*, was identified in field populations of the DBM resistant to chlorantraniliprole. The high expression of this gene was found to mediate the resistance of the DBM to various insecticides. DBM resists the toxicity of insecticides through two distinct mechanisms: direct degradation and counteracting oxidative stress mediated by the regulation of *PxGSTS1*. Further miRNA target prediction revealed that pxy-miR-8528a targets the 3′UTR of *PxGSTS1*, inhibiting its expression. *In vitro* and *in vivo* experiments further demonstrated the relationship between pxy-miR-8528a and *PxGSTS1*. Expression profile results showed that pxy-miR-8528a was highly expressed in the field-resistant populations. Field populations injected with agomir-8528a exhibited increased sensitivity to chlorantraniliprole and a significant increase in the insecticide-induced accumulation of various types of reactive oxygen species (Liu et al., 2024). Additionally, *GSTs4* was highly over-expressed in the indoxacarb-resistant strain of FAW. Dual-luciferase reporter assays demonstrated the target relationship between miR-317-3p, miR-283-5p and *GSTs4*. Injection of miR-317-3p and miR-283-5p agomirs reduces the expression level of *GSTs4* and increases the susceptibility of FAW to indoxacarb. Conversely, injection of miR-317-3p and miR-283-5p antagomirs increases *GSTs4* expression and reduces the larval susceptibility to indoxacarb (Wu et al., 2024).

*Tetranychus cinnabarinus* is a significant agricultural pest mite with a broad range, feeding on over 100 plant species. Chemical agents and acaricides are currently the primary methods used to control this mite (Jia et al., 2011). Cyflumetofen, a benzoyl acetonitrile acaricide, was first registered in Japan in 2007 and has shown effective control against various mite species (Zhou et al., 2020). The main targets of cyflumetofen include CarE, mixed-functional oxidase (MFO), GSTs, and esterases (Wang et al., 2014; Wei et al., 2016a). In the context of resistance, miR-1-3p was found to be downregulated in cyflumetofen-resistant strains of *Tetranychus cinnabarinus*. This miRNA specifically targets the detoxification enzyme gene GST (*TCGSTM4*, a mu-class GST gene) (Zhang et al., 2018). Overexpression of miR-1-3p results in a significant downregulation of the *TCGSTM4* gene and decreases the sensitivity of both cyflumetofen-resistant and -sensitive strains of *T. cinnabarinus* to cyflumetofen. Conversely, reducing the expression of miR-1-3p enhances the sensitivity of both resistant and sensitive strains to cyflumetofen (Zhang et al., 2018).

**FIGURE 1:**
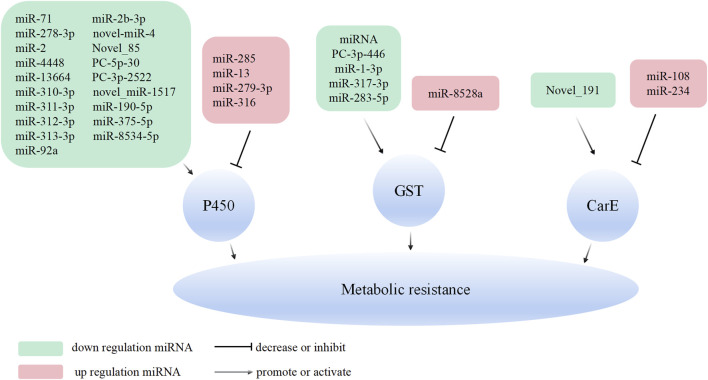
Overview of the molecular mechanisms of miRNAs in metabolic resistance. MiRNA can bind to the 3′UTR or CDS of the target gene (P450, GSTs, and CarE) and inhibit its expression.

In conclusion, the research underscores the pivotal role of P450 enzymes, CarE, and GSTs in the development of metabolic resistance in various insect species, highlighting the complexity and diversity of regulatory mechanisms involved. The up/down regulated miRNAs specifically target the detoxification enzyme genes such as P450, CarE, and GSTs. This, in turn, modulates the expression of detoxification enzyme genes, which are involved in the development of metabolic resistance in insects and mites (Figure 1). This comprehensive understanding of the genetic and post-transcriptional regulatory mechanisms underlying insecticide resistance can inform the development of more effective pest management strategies and novel insecticides.

### 3.3 Involvement of miRNAs in the regulation of target-site resistance

miRNAs play a crucial role in regulating target-site resistance by primarily exerting post-transcriptional control over insecticide target genes. These target genes encompass a range of important molecular targets, including acetylcholinesterase (*AChE*), voltage-gated sodium channel (*VGSC*), nicotinic acetylcholine receptor (*nAChR*) and ryanodine receptor (*RyR*) (Siddiqui et al., 2022). To provide an overview of the regulatory interactions, Table 2 presents a summary of miRNAs that target different insecticide target genes, ultimately contributing to the modulation of insect resistance to various insecticides.

**TABLE 2:** List of insect microRNAs involved in target-site resistance to different insecticides.

Species	MicroRNA	Target gene	miRNA regulation	Exposure	Year	References
*Aedes aegypti*	miR-33	*VGSC*	Up	Permethrin	2021	Kubik et al. (2021)
*Plutella xylostella*	miR-7a miR-8519	*RyR*	Down	Chlorantraniliprole	2015	Li et al. (2015)
	miR-998-3p	*ABCC2*		Cry1Ac	2020	Zhu et al. (2020)
	miR-8510a-3p	*PxABCG3*		Cry1Ac	2024	Yang et al. (2024)
	novel-miR-310	*PxABCG20*	UP	Cry1Ac	2022	Yang et al. (2022a)
	miR-2b-3p	*PxTrypsin-9*		Cry1Ac	2024	Zhang et al. (2024)
	miR-189942	*EcR-B*		Fufenozide	2020	Li et al. (2020)
*Chilo suppressalis*	miR-7322-5p	*p38*	Down	Cry1Ca	2023	Wu et al. (2023)
*Aphis gossypii*	miR-276	*ACC*	Down	Spirotetramat	2016	Wei et al. (2016b)
	miR-3016					
*Sitobion miscanthi*	miR-278	*nAChRα1A*	Up	Imidacloprid	2021	Zhang et al. (2021a)
	miR-263b	*nAChRβ1*			2022	Zhang et al. (2022c)
*Nilaparvata lugens*	miRNA novel_268	*NlABCG3*	Down	Nitenpyram and Clothianidin	2022	Li et al. (2022)
*Laodelphax striatellus*	PC-5p-30_205949	*ABCG23*	Down	Triflumezopyrim	2023	Wang et al. (2023a)
	PC-5p-3096_674	*ABCA3*			2022	Yang et al. (2022b)
*Leptinotarsa decemlineata*	miR-12-1-5p	*ADAM10*	Up	Cry3Aa	2022	Robles-Fort et al. (2022)
	miR-12-2-5p					
	miR-252a-5p					
	miR-2796-5p					
	miR-316-5p					
	miR-998-5p					

*Aedes aegypti* is susceptible to pyrethroids and DDT, with *voltage-gated sodium channels* (*VGSC*) serving as the primary target (Du et al., 2013). In contrast, miR-33, targets *VGSC*. Overexpression of miR-33 in both permethrin-resistant and sensitive strains of *A. aegypti* downregulates the *VGSC*, gene expression, leading to reduced mortality caused by permethrin (Kubik et al., 2021).

Insecticide target receptor genes, particularly ryanodine receptors (*RyRs*), are involved in conferring in insect resistance to chlorantraniliprole. Studies conducted on DBM have demonstrated that field-collected resistant lines displayed 2.28–4.14 times greater *RyR* expression compared to chlorantraniliprole-sensitive lines. Following a 12-hour exposure to chlorantraniliprole at LC_50_ and LC_75_ concentrations, there was a 5-fold and 7.2-fold upregulation in *RyR*, respectively. Subsequent investigations have unveiled that miR-7a and miR-8519 selectively target the 3′UTR region of the *RyR* gene, leading to the suppression of its expression and exerting an impact on the sensitivity of DBM to chlorantraniliprole (Li et al., 2015).

Fufenozide is a dibenzoylhydrazine-type non-steroidal ecdysone agonists, functions by targeting insect ecdysone receptors (*EcR*), primarily for the control of Lepidopteran insects (Sun et al., 2010). The 3′UTR of the DBM *EcR-B* gene is targeted by miR-189942, and its over-expression significantly suppresses *EcR-B* gene expression. Consequently, the increased expression or knockout of miR-189942 modulates the sensitivity of DBM to fufenozide, resulting in enhanced tolerance of DBM to this insecticide (Li et al., 2020). In *A*. *gossypii*, both miR-276 and miR-3016 act together to upregulate the expression of acetyl-CoA carboxylase (ACC), which plays a crucial role in conferring resistance to spirotramat in *A. gossypii* (Wei et al., 2016b). Furthermore, miR-263b and miR-278 in *S. miscanthi* are involved in mediating resistance to imidacloprid by suppressing the expression of insecticide receptor genes, specifically *nAChRβ1* and *nAChRα1A* genes, respectively (Zhang et al., 2021a; Zhang et al., 2022c).

The receptor gene encoding ABC transporter proteins of Bt Cry proteins can be classified into eight subfamilies ranging from ABCA to ABCH (Dean and Annilo, 2005). Among these subfamilies, ABCA, ABCB, ABCC, ABCD and ABCG have been implicated d in resistance to various insecticides (Labbe et al., 2011). Analysis of miRNA in Cry1Ac-resistant and sensitive DBM strains has revealed the involvement of miRNA in resistance development by targeting the coding sequence (CDS) region of ABC transporters (Yang et al., 2021a). In particular, *PxABCG20* and *PxABCG3*, which exhibit differential expression between the two strains, are targeted by novel-miR-310 and miR-8510a-3p, respectively. Upregulation of novel-miR-310 and miR-8510a-3p expression suppresses the expression of *PxABCG20* and *PxABCG3*, respectively, thereby enhancing the tolerance to Cry1Ac in the sensitive strain. Conversely, by suppressing novel-miR-310 and miR-8510a-3p, the upregulation of *PxABCG20* and *PxABCG3* occurs, leading to a reduction in Cry1Ac tolerance (Yang et al., 2022a; Yang et al., 2024). Furthermore, miR-998-3p targets the CDS region of *ABCC2*, which is conserved across three lepidopteran insects: *Helicoverpa armigera*, *Spodoptera exigua*, and DBM. Injection of miR-998-3p agomir results in a reduction of *ABCC2* expression, thereby enhancing tolerance to Cry1Ac in all three insects. Conversely, injection of miR-998-3p antagomir increases the expression of *ABCC2*, reducing the resistance of Cry1Ac-resistant DBM strains to Cry1Ac (Zhu et al., 2020). After treating with sublethal concentrations of Cry1Ac, the *PxTrypsin-9* gene exhibits high expression in third instar DBM larvae, and silencing *PxTrypsin-9* significantly reduces Cry1Ac toxicity in DBM. Both *in vitro* and *in vivo* experiments have demonstrated that miR-2b-3p targets the CDS region of *PxTrypsin-9*, suppressing its expression and enhancing Cry1Ac tolerance in DBM (Zhang et al., 2024).

*p38*, *xbp-1*, and *ire-1* have been identified as receptor genes for Cry toxins. Interfering with the expression of these genes, enhances the effect of Cry toxins on the sensitivity of different insects such as *C. suppressalis*, *Aedes aegypti*, and *Manduca sexta* (Cancino-Rodezno et al., 2010; Bedoya-Perez et al., 2013; Qiu et al., 2017). In the case of *C. suppressalis*, further research have shown that miR-7322-5p is downregulated in insects that were fed Cry1Ca transgenic rice. This miRNA targets 3′UTR of *p38*, inhibiting its expression and negatively regulating the sensitivity of stem borers to Cry1Ca. Feeding on Cry1Ca enhances the phosphorylation of the Hsp19 protein in stem borers, which is downstream of *p38*. The downregulation of *hsp19* gene expression increases the sensitivity of *C. suppressalis* to Cry1Ca, suggesting that the activation of *hsp19* through the miR-7322-5p/p38/Hsp19 pathway promotes the sequestration of Cry1Ca in *C. suppressalis* (Wu et al., 2023). While most Cry1 genes are effective against lepidopteran insects, Cry3 protein primarily acts on coleopteran insects (Jouzani et al., 2017). The interaction between Cry3Aa protein and *ADAM10* metalloprotease, located on the midgut membrane, is crucial for the toxin’s function in Colorado potato beetle larvae (Ruiz-Arroyo et al., 2017). In Colorado potato beetle larvae, lde-miR-12-1-5p, lde-miR-12-2-5p, lde-miR-252a-5p, lde-miR-2796-5p, lde-miR-316-5p, and lde-miR-998-5p target *ADAM10* and may be involved in regulating susceptibility to Cry3Aa (Robles-Fort et al., 2022).

**FIGURE 2:**
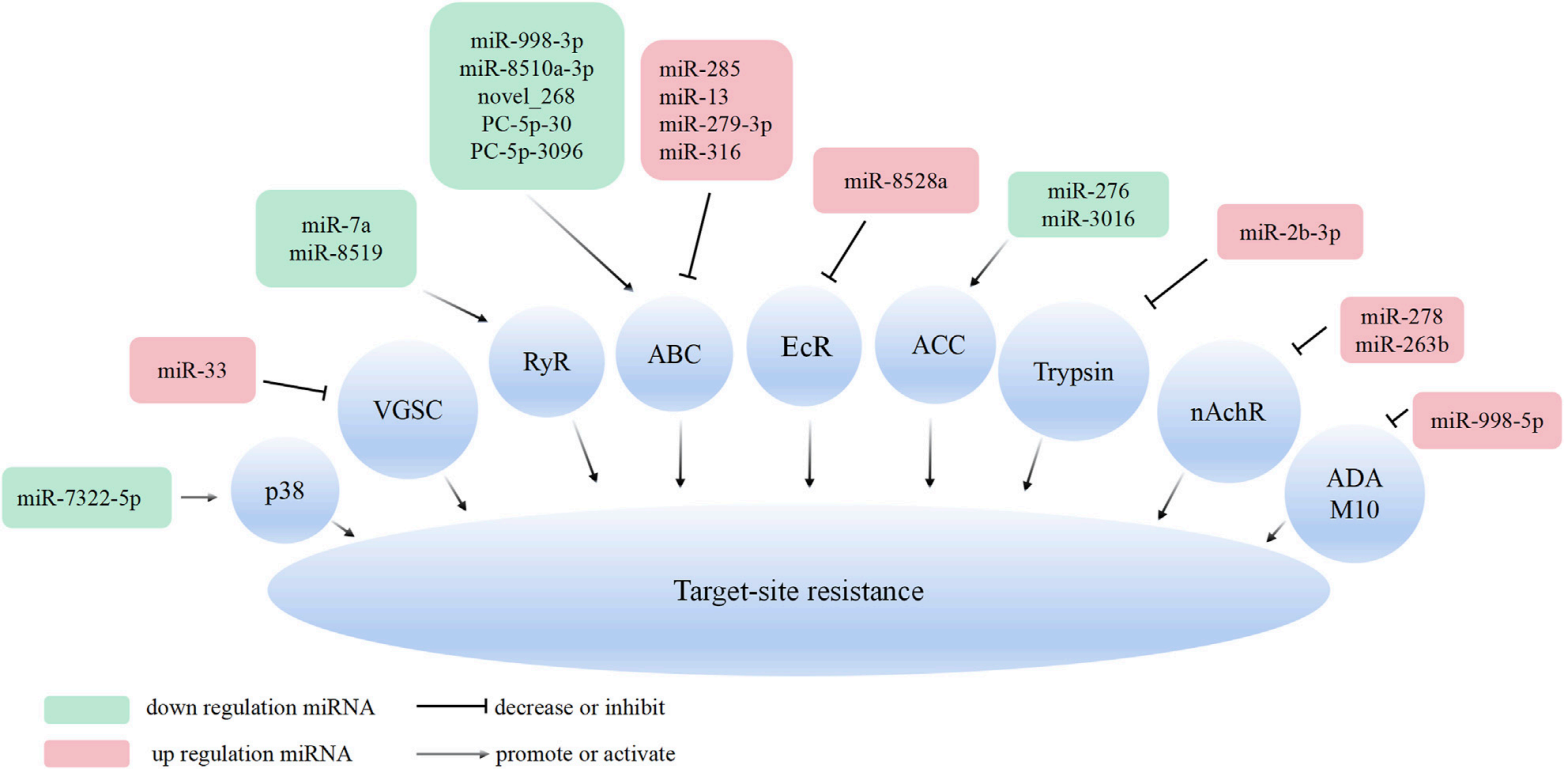
Overview of the molecular mechanisms of miRNAs in insect target-site resistance. MiRNA can bind to the 3′UTR or CDS of the insecticide target gene and inhibit its expression.

Similar to the miRNA in the regulation of metabolic resistance, miRNAs conferred in the target-site resistance were closely related to miRNA regulation of insecticide target gene expression (Figure 2).

## 4 LncRNA is involved in regulating insecticide resistance development

The development of insecticides resistance in insect is closely linked to the highly expression of lncRNA genes in resistant strains. Transcriptome analysis of resistant and sensitive strains of DBM exposed to various insecticide, including Bt, chlorpyrifos, fipronil, and deltamethrin, have revealed the association of specific lncRNAs with resistance. For instance, Lawrie et al. (2021) found 358 highly expressed lncRNAs associated with chlorpyrifos resistance, 280 lncRNAs associated with fipronil resistance, and 59 lncRNAs associated with Bt resistance. Similarly, Wang et al. (2023b) observed differential expression of multiple lncRNAs in FAW larvae treated with 23 pesticides. In another study, Wu et al. (2022) demonstrated the differential expression of a significant number of lncRNAs in *Tribolium castaneum* exposed to terpinene-4-ol. Cotton bollworm resistant to Bt also exhibited the presence of multiple highly expressed lncRNA genes (Rahman et al., 2024). Notably, the lncRNA gene lnc377.4 was found to be highly expressed in malathion-resistant *Bactrocera dorsalis* strains, and its downregulation increased susceptibility to malathion (Meng et al., 2021). Similarly, highly expressed lncRNAs LNC_004867 and LNC_006576 were closely associated with the development of indoxacarb resistance in *Spodoptera litura* (Shi et al., 2022). Similar observations were made in chlorantraniliprole-resistant DBM strains, where the high expression of lncRNAs TCONS_00013329 and TCONS_00056155 was associated with the expression of the RyR gene, contributing to resistance development (Zhu et al., 2017b). In chlorantraniliprole-resistant and sensitive strains of *C. suppressalis*, the identification of 3,470 lncRNAs led to the discovery that the high expression of lncRNA MSTRG.7482.1 contributes to chlorantraniliprole resistance, as demonstrated by RNAi and bioassay experiments (Huang et al., 2022).

**FIGURE 3:**
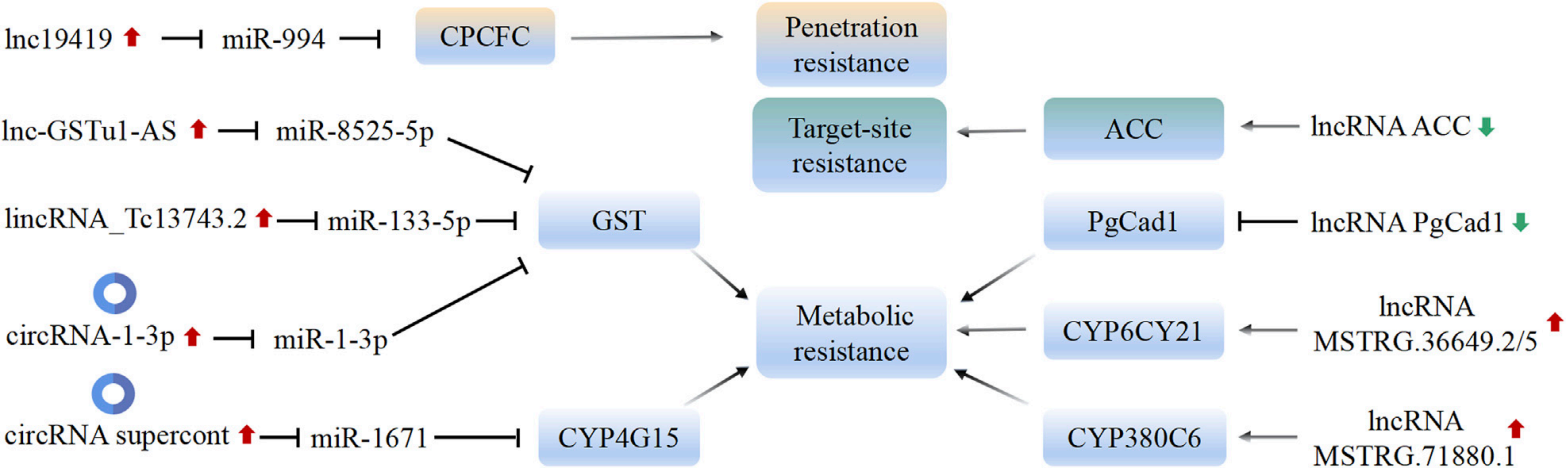
Mechanism of insecticide resistance mediated by lncRNA and circRNA in insects. The common mechanisms by which lncRNA/circRNA affecting insecticide resistance include: 1) acting as a sponge of miRNAs, which affects the expression of target genes; 2) influencing the expression of target or downstream genes. Black arrows represent promotion or activation; black blunt arrows represent decrease or inhibition. The green or red arrows indicate whether the function of lncRNA/circRNA in insects promotes or inhibits the insecticide resistance.

In-depth research suggests that lncRNA genes primarily mediate the development of insect resistance through the regulation of detoxification enzyme genes and insecticide target genes (Figure 3). For instance, Li S. et al. (2019a) demonstrated the influence of the lncRNA PgCad1 on the susceptibility of *Pectinophora gossypiella* to the Bt toxin protein Cry1Ac by regulating the expression of the *PgCad1* gene. Similarly, Shen et al. (2021) identified 25 lncRNAs in *Phyllotreta striolata* that target detoxification enzyme genes. In the spirotetramat-resistant strain of *A*. *gossypii*, the highly expressed detoxification enzyme genes CYP4CJ1, CYP6CY7, and CYP6CY21 play a significant role. Down-regulation of these genes increases the mortality of nymphs and adults in the resistant strain. Peng et al. (2022) found that silencing of lncRNAs MSTRG.36649.2/5 and MSTRG.71880.1 alters the expression of *CYP6CY21* and *CYP380C6*, which in turn affects the susceptibility of the resistant *A. gossypi*i strain to spirotetramat. This suggests that these two lncRNAs are involved in the development of spirotetramat resistance in *A. gossypii* by regulating detoxification enzyme genes. Furthermore, lncRNA ACC contributes to spirotetramat resistance in *A. gossypii* by regulating ACC transcription (Peng et al., 2021).

In-depth research on lncRNAs in insects has revealed one mechanism by which insecticide resistance is regulated, namely, the up-regulation or down-regulation of lncRNAs, which in turn leads to the up-regulation or inhibition of target or downstream genes (Figure 3). However, there is an exception with the lncRNA ACC. The insecticidal mechanism of spirotetramat involves the inhibition of *ACC* expression (Nauen et al., 2008). Interestingly, over-expression and novel mutations in the *ACC* gene have been found to contribute to high spirotetramat resistance in A. gossypii (Pan et al., 2017). Furthermore, the downregulation of the lncRNA ACC has been observed in spirotetramat-resistant *A. gossypii*, which in turn affects the expression of genes related to *ACC* (Peng et al., 2021). Additionally, two transcription factors, C/EBP and C/EBPzeta, have been found to regulate the transcription level of the lncRNA ACC (Peng et al., 2021). In cancer research, the mechanisms of therapy resistance mediated by lncRNAs often involve direct interactions with transcription factors or RNA-binding proteins, thereby affecting the expression of downstream genes and pathways (Singh et al., 2022). The specific mechanisms by which transcription factors bind to and regulate the expression of lncRNAs, as well as the interactions between lncRNAs and proteins, remain to be explored in insects.

## 5 Role of lncRNA/circRNA-miRNA-mRNA(ceRNA) regulatory networks in the development of insecticide resistance

The competing endogenous RNA (ceRNA) mechanism was initially discovered in pseudogenes, which possess highly conserved microRNA response elements (MREs) that can bind to miRNA along with protein-coding genes. Pseudogenes act as decoys, attracting miRNA binding and reducing the inhibitory effect of miRNA on mRNA translation (Poliseno et al., 2010). In theory, any RNA molecule containing miRNA binding sites can function as a ceRNA. Therefore, ceRNA can include pseudogenes, protein-coding transcripts, lncRNAs, and circRNAs (Wang et al., 2016). It has been demonstrated that the over-expression of lncRNAs is essential for their “sponge” function and initiation of the ceRNA regulatory pathway. For example, the over-expressed lncRNA-NEAT1 acts as a “sponge” for miR-377-3p, thereby promoting the expression of *E2F3* (Sun et al., 2016). CircRNAs are a recent addition to the ceRNA mechanism and their unique characteristics make them well-suited for ceRNA formation. These characteristics include a lack of free ends, predominant cytoplasmic localization, and a predominantly non-coding nature (Wang et al., 2016). While the ceRNA mechanism has been extensively studied in human diseases and cancers, its regulatory networks in insects remain relatively unexplored (Fan et al., 2023; Meng et al., 2023; Ren et al., 2023). In the chlorantraniliprole-resistant DBM strain, both the *GSTu1* gene and its anti-sense transcript, lnc-GSTu1-AS, are upregulated. lnc-*GSTu1*-AS forms a double-stranded duplex with GSTu1, occupying the miR-8525-5p binding site in the *GSTu1*-3′UTR. This prevents miR-8525-5p-induced degradation of *GSTu1*, thereby maintaining *GSTu1* mRNA stability and contributing to the development of resistance to chlorantraniliprole in DBM (Zhu et al., 2021).

The research conducted by Meng et al. (2023) suggests that in *B. dorsalis*, the lnc19419-miR-994-CPCFC ceRNA regulatory network plays a role in regulating the penetration resistance to malathion. The injection of miR-994 leads to an increase in the thickness of the epidermis in *B. dorsalis*, enhancing its tolerance to malathion. This effect is achieved through the targeting of the *CPCFC* gene by miR-994. Additionally, the lnc19419 molecule is shown to upregulate the expression of the *CPCFC* gene in the malathion-resistant strain by adsorbing miR-994. Overall, this lnc19419-miR-994-CPCFC ceRNA regulatory network influences the penetration resistance of *B. dorsalis* to pesticides by modulating the epidermal thickness.

The studies conducted by Lv et al. (2022) and Feng et al. (2020, 2022) provide evidence for the involvement of circular RNAs and long intergenic non-coding RNAs (lincRNAs) in regulating insecticide resistance in different insect species. In the deltamethrin-resistant strains of *C. pipiens*, the circular RNA supercont3.352:252102|253283 is upregulated. This circRNA acts as a sponge for miR-1671, inhibiting its activity and leading to the down-regulation of the *CYP4G15* gene. This regulatory mechanism plays a role in regulating the resistance of *C. pipiens* to deltamethrin (Lv et al., 2022). Similarly, in cyflumetofen-resistant strains of *Tetranychus cinnabarinu*s, the lincRNA_Tc13743.2 functions as a ceRNA by binding to miR-133-5p. This interaction regulates the expression of the *TcGSTm02* gene, which is involved in cyflumetofen resistance (Feng et al., 2020). Furthermore, the circRNA-1-3p in *T. cinnabarinus* also acts as a ceRNA by sequestering miR-1-3p. This interaction promotes the over-expression of the *TcGSTm02* gene, contributing to the resistance of *T. cinnabarinus* to cyflumetofen (Feng et al., 2022).

The results from the studies mentioned in the previous conversation support the notion that the regulation of insecticide penetration and metabolic resistance in insects involves a cascade of interactions between lncRNAs/circRNAs, miRNAs, and mRNAs. In this regulatory cascade, the abundant lncRNAs/circRNAs act as competitive sponges for miRNAs, preventing their negative interaction with cuticle protein or detoxification enzyme genes and ultimately leading to increased expression of these genes (Figure 3). For cuticle protein, the accumulated protein in the cuticle forms a complex with synthesized chitin, resulting in the thickening of the pronotum cuticle. This thickened cuticle serves as a more effective barrier against insecticide penetration, thus contributing to increased insecticide resistance (Meng et al., 2023). In the case of detoxification enzymes, the over-expression of lncRNAs/circRNAs promotes the expression of these enzymes by sequestering miRNAs. This regulatory mechanism plays a role in the evolution of insecticide resistance in insects. Overall, this regulatory cascade involving lncRNAs/circRNAs, miRNAs, and mRNAs contributes to the modulation of insecticide penetration and metabolic resistance in insects (Figure 3); (Feng et al., 2020; Feng et al., 2022; Lv et al., 2022).

## 6 Conclusion and future perspectives

Investigating the role of regulatory factors and ncRNAs in the development of insecticide resistance is indeed crucial. Understanding the molecular mechanisms by which ncRNAs regulate insect resistance provides valuable insights for pest resistance research. In this article, the role of miRNAs, lncRNAs, and the lncRNA/circRNA-miRNA-mRNA (ceRNA) regulatory network in regulating insecticide resistance in insects is reviewed. One primary mechanisms by which ncRNA regulate insect resistance is through the modulation of target genes such as *RyR*, *VGSC*, and *nAchR*. By regulating the expression of these target genes, miRNAs can influence insect susceptibility to chemical pesticides. Another way ncRNAs contribute to insect resistance is by up-regulating the expression of detoxification metabolism enzyme genes, such as P450, GSTs, and CarE, which leads to increased metabolism of chemical pesticides. The ceRNA regulatory network, involving lncRNA/circRNA-miRNA-mRNA interactions, has been identified as a regulator mechanisms in insects resistance. This network influences the expression of detoxification enzyme genes, such as P450 and GSTs, thereby affecting the insect’s metabolic resistance. Additionally, changes in cuticle thickness, regulated by this network, can influence the penetration resistance of insects to insecticides (Figures 1–3).

The majority of protein-coding genes are likely regulated by miRNAs (Ha and Kim, 2014), and miRNA have unique functions. miRNAs have the capacity to regulate the expression of multiple genes within the signaling pathway, both upstream and downstream (Krol et al., 2010). The development of insect resistance to insecticides involves co-regulation of multiple genes. For instance, after feeding on Bt pathogens, JH/20E in the midgut epithelium of DBM binds to the respective Met-Tai and EcR-USP heterodimeric receptor complexes, activating the key gene of the downstream of MAPK signaling pathway. As a result, *MAP4K4* orchestrates the significant up-regulation of the transcription factors GATAd and *PxmALP* genes while inversely regulating the differential expression of the midgut receptor genes *ALP* and *ABCC* (*ABCC2* and *ABCC3*). This intricate process ultimately leads to the development of high resistance to Cry1Ac in the DBM without affecting its growth and development (Guo et al., 2015; Guo et al., 2020; Guo et al., 2022). However, the current study only found that miR-998-3p as binding to the CDS region of *ABCC2* and regulating the development of resistance to Cry1Ac in DBM (Zhu et al., 2020). It remains unclear whether other miRNAs are involved in the MAPK signaling pathway or if miR-998-3p regulates the expression of other genes within the pathway. Despite the fact that individual miRNAs have been shown to modulate various aspects of insecticide resistance, our understanding of the intricate interactions within these regulatory pathways remains limited. Furthermore, the dysregulation of miRNA expression has been observed in insects under insecticide stress, with most characterized by downregulated expression (Tables 1, 2). The regulatory mechanism underlying this down-regulated expression are yet to be fully elucidated. Additionally, studies on insect lncRNAs and circRNAs are still primarily focused on gene sequence mining, with the poor conservation of lncRNAs and circRNA among different species posing a significant hindrance. This limitation further restricts our understanding of the functions and mechanisms of lncRNAs and circRNAs (Ebbesen et al., 2017; Choudhary et al., 2021). Given the lncRNA/circRNA-miRNA-mRNA (ceRNA) regulatory network has been implicated in the insecticide resistance, integrating multi-omics approaches, such as transcriptomics, proteomics, and metabolomics can provide a systems-level perspective on the regulatory mechanism of ncRNAs in responses to insecticide exposure (Feng et al., 2020; Feng et al., 2022; Lv et al., 2022; Meng et al., 2023). By combining data from multiple platforms, researchers can unravel complex regulatory networks, identify upstream regulators for lncRNAs and circRNAs, and elucidate downstream signaling pathways involved in the development of insecticide resistance (Muthu Lakshmi Bavithra et al., 2023). Future research should prioritize systematic cross-species comparisons to elucidate the evolutionary dynamics of ncRNA-mediated resistance mechanisms. Comparative genomics analyses across diverse insect species can unveil conserved ncRNA regulatory networks and identify novel targets for managing insecticide resistance.
